# Long‐term outcome of Wilson's disease complicated by liver disease

**DOI:** 10.1002/jgh3.12589

**Published:** 2021-06-05

**Authors:** Satoko Arai, Tomomi Kogiso, Yuri Ogasawara, Takaomi Sagawa, Makiko Taniai, Katsutoshi Tokushige

**Affiliations:** ^1^ Department of Internal Medicine, Institute of Gastroenterology Tokyo Women's Medical University Tokyo Japan

**Keywords:** acute liver failure, aspartate aminotransferase to platelet ratio index, fibrosis‐4 index, liver transplantation, Wilson's disease

## Abstract

**Background and Aim:**

Wilson's disease (WD) is a rare inherited disease that causes systemic copper accumulation. This study examined the long‐term course of WD patients with liver disease.

**Methods:**

The 12 patients (9 female patients) enrolled in the study had a median age of 28 years (range: 19–57 years) at their first visit to our hospital. Clinical course and fibrosis markers were assessed in all patients.

**Results:**

The median age at diagnosis was 24 years (range: 5–42 years). One patient had acute liver failure (ALF) and 11 patients had chronic liver disease (CLD, 5 with cirrhosis). The patients were followed‐up for >20 years. The patient with ALF underwent liver transplantation; the postoperative course during the subsequent 20 years was good. Of the six patients with CLD, liver cirrhosis developed in four patients with interrupted chelating therapy. Two of the patients with cirrhosis died; one of these two patients died at 21 years after liver transplantation. However, the remaining patients with continued treatment exhibited a favorable clinical course for 30 years and none developed hepatocellular carcinoma (HCC). The duration of chelation therapy was significantly negatively correlated (*P* < 0.05) with the fibrosis‐4 index or aspartate aminotransferase to platelet ratio index (APRI) score at the last visit; lower values were indicative of greater treatment success. Patients with an APRI score ≥1.5 had a significantly worse prognosis (*P* < 0.05).

**Conclusion:**

Long‐term survival of patients with WD was achieved without worsened liver function or carcinogenesis with appropriate treatment. Treatment disruption should be avoided.

## Graphical abstract

The duration of chelation therapy for Wilson's disease complicated by liver disease was significantly negatively correlated with the fibrosis‐4 index and aspartate aminotransferase to platelet ratio index (APRI) score at the last visit.

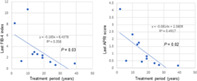

## Introduction

Wilson's disease (WD) is a rare genetic disorder of copper metabolism that usually manifests during childhood, adolescence, or early adulthood.^1^ It is caused by mutations in the gene encoding a copper‐transporting P‐type ATPase (ATP7B)^2^, ^3^, ^4^ located in the trans‐Golgi network of hepatocytes. The reported prevalence of WD is 1 in 30 000 in the general population, including in Japan.^4^, ^5^ The key features of WD are liver disease, neurologic symptoms, and Kayser–Fleischer (K–F) rings. A scoring system for WD—established in 2001—enables diagnosis based on symptoms and laboratory tests.^6^ Liver disease typically precedes neuronal manifestations by 10 years. WD with liver disease is wide‐ranging in its manifestations, from asymptomatic to overt cirrhosis and acute liver failure (ALF), chronic liver disease (CLD), and cirrhosis. In the case series reported by Ferenci et al.,^6^ 30 of 64 patients had liver disease; 17% of the patients had ALF. Whereas ALF, K–F rings, and neurological abnormalities are absent in most patients with WD, hemolysis and acute renal failure are common. The disease progresses rapidly and can lead to “fulminant” liver failure.

Medical treatment for WD with liver disease currently consists of chelating agents (d‐penicillamine and trientine) and zinc acetate dihydrate^7^, ^8^; these liver disease‐controlling treatments protect against fibrosis development and improve patient prognosis. Patients with liver failure receive liver transplantation (LT).

Studies evaluating fibrosis in patients with CLD largely rely on noninvasive markers, such as the fibrosis‐4 (FIB‐4) index^9^ and the aspartate aminotransferase (AST) to platelet (PLT) ratio index (APRI).^10^ Recently, the efficacy of these fibrosis assessments was demonstrated in patients with WD, such that an FIB‐4 index <3.25 and an APRI score <1.5 were sufficient to rule out cirrhosis with specificities of 95% and 93%, respectively.^11^ However, a long‐term follow‐up study has not yet been reported.

In this study, we evaluated changes in fibrosis based on noninvasive markers, as well as the patient prognosis, among patients with WD and liver disease who attended follow‐up examinations for a median of 20 years. The study was conducted in accordance with the principles of the Declaration of Helsinki and the ethical guidelines of Tokyo Women's Medical University Hospital (Tokyo, Japan).

## Methods

### Patients and study design

Of the 15 patients with WD who were treated at our hospital, long‐term follow‐up data were available for 12 patients (3 male patients and 9 female patients). The median patient age at the initial visit to our hospital was 28 years (range: 19–57 years). Six patients had a family history of WD.

WD was diagnosed based on clinical signs (e.g., K–F rings and neurologic symptoms), biochemical test results (e.g., copper levels in serum, urine, and liver, as well as serum ceruloplasmin), and genetic test findings.^12^, ^13^ Liver cirrhosis and hepatobiliary malignancies were diagnosed according to imaging manifestations, biochemical results, and/or pathological findings in the liver.

In our patients, the following data were obtained: baseline characteristics (age, sex, history, and complications of liver disease); serum levels of albumin (ALB, g/dL), total bilirubin (T‐BIL, mg/dL), direct bilirubin (D‐BIL, mg/dL), AST (U/L), alanine aminotransferase (ALT, U/L), and gamma‐glutamyltransferase (GGT, U/L); ammonia (NH_3_, μg/dL); prothrombin time (PT, %) and PT‐international normalized ratio (INR); white blood cell count (WBC, /μL); and PLT count (×10^4^/μL). Serum levels of copper (μg/dL) and ceruloplasmin (g/L), as well as urinary copper excretion (μg/day), were retrieved from the clinical charts. Fibrosis was estimated based on the FIB‐4 index, defined as: age in years × AST (U/L)/PLT (10^9^/L) × (ALT [U/L] 1/2).^9^ Fibrosis was also estimated based on the APRI score: 100 × (AST level/upper limit of normal)/PLT [10^9^/L]).^10^ Liver function was evaluated by calculating the model for end‐stage liver disease (MELD) score ^14^ and a prognostic score (total score of serum bilirubin, AST, INR, WBC, and ALB).^15^

Of the patients in this study, four had been treated with zinc acetate dihydrate, three had been treated with d‐penicillamine, nine had been treated with trientine dihydrochloride, and four had been treated with pyridoxal phosphate hydrate at the discretion of the attending physician.

### Statistical analysis

The data are presented as medians, with minimum and maximum values. Differences were considered statistically significant based on a *P* value of <0.05. Changes of the FIB‐4 index and APRI score were evaluated; significant differences were estimated using the Mann–Whitney *U*‐test in SPSS Statistics (version 25.0; IBM Corp., Armonk, NY, USA). The FIB‐4 index, APRI score, and period of treatment with chelating agents were plotted in scatter plots. The results were evaluated based on the approximated line and the *R* ^2^. The survival rate was assessed by constructing Kaplan–Meier curves using SPSS Statistics. The survival rate according to type of onset, FIB‐4 index, and APRI score was estimated using a log‐rank test. The threshold value of the FIB‐4 index was 2.67, in accordance with the non‐alcoholic fatty liver disease (NAFLD)/non‐alcoholic steatohepatitis (NASH) guideline,^16^ and the threshold value of the APRI score was 1.5.^17^

## Results

### Characteristics of patients with WD

The median age at diagnosis of the nine female and three male patients was 24 years (range: 5–42 years), and the median duration of follow‐up was 20 years (range: 10–39 years) (Table 1). Presentation was triggered by general malaise in two patients, liver damage in three patients, a family search due to the sibling‐onset of WD in four patients, and WD‐related findings in three patients. One patient had ALF and the remaining 11 patients had CLD, including five with liver cirrhosis. Concomitant findings were K–F corneal rings in six patients and neurological and psychological symptoms (e.g., dysarthria) in two patients each. Patients were referred to our hospital at a median age of 28 years (range: 19–57 years). At that time, their serum values were as follows: ALB, 3.5 g/dL (range: 1.9–4.8 g/dL); T‐BIL, 0.9 mg/dL (range: 0.2–40.8 mg/dL); AST, 34 U/L (range: 15–107 U/L); ALT, 24 U/L (range: 7–135 U/L); GGT, 61 U/L (range: 15–188 U/L); PT, 56.1% (range: 28.9–100.0%); PT‐INR, 1.29 (range: 0.90–2.01); and PLT count, 15.4 × 10^4^/μL (range: 2.6–25.7 × 10^4^/μL). Their serum copper, serum ceruloplasmin, and urinary copper levels were 27.0 μg/dL (range: 5.0–214.0 μg/dL), 4 mg/dL (range: 2–14 mg/dL), and 131.8 μg/day (range: 2.0–14 965.0 μg/day), respectively. The copper content in liver tissue (measured in four patients) was 687.8 μg/g dry weight (range: 359.0–1130.8 μg/g dry weight). Four patients also had esophageal/gastric varices. The MELD and prognostic scores were high in the patient with ALF (#3) and in a patient (#10) with end‐stage liver failure (Table S1, Supporting information). The pathological findings of the liver showed steatohepatitis (Table 2).

**Table 1:** Baseline characteristics and biochemical data of the 12 study patients with Wilson's disease at first visit or diagnosis at our hospital

Cases	*n* = 12
Age at diagnosis (years)	24 (5–42)
Age at referral to our hospital (years)	28 (19–57)
Duration from diagnosis to last observation (years)	20 (10–39)
Sex (M, male; F, female)	M: 3 cases, F: 9 cases
Acute /chronic	1 case of 11 cases
K–F ring	7 cases (1 case observed during follow‐up)
Neuronal symptoms	2 cases
Psychiatric symptoms	2 cases
Esophageal varices	4 cases (3 cases observed during follow‐up)
Familial onset	Brother 3 cases, Sister 7 cases
Initial diagnosis of liver cirrhosis	5 cases
Biochemical analysis at age at referral to our hospital	
ALB (g/dL)	3.5 (1.9–4.8)
T‐/D‐BIL (mg/dL)	0.9 (0.2–40.8)
AST (U/L)	34 (15–107)
ALT (U/L)	24 (7–135)
GGT (U/L)	61 (15–188)
Copper (μg/dL) (*n* = 9)	27.0 (5.0–214.0)
Ceruloplasmin (mg/dL) (*n* = 10)	4 (2–14)
U‐copper (μg/day) (*n* = 9)	131.8 (2.0–14 965.0)
NH3 (μg/dL) (*n* = 5)	45 (10–109)
PT (%)/INR	56.1 (28.9–100.0)/1.29 (0.90–2.01)
WBC (/μL)	4840 (3020‐14 900)
PLT (×10^4^/μL)	15.4 (2.6–25.7)
MELD score	5.5 (1.0–28.0)
Prognostic index	1 (0–11)
Treatment	
Zinc acetate dihydrate	4 cases
Trientine dihydrochloride	9 cases
d‐penicillamine	3 cases
Pyridoxal phosphate hydrate	4 cases
Discontinued treatment	4 cases
Self‐interruption of treatment	4 cases
Length of discontinuation hospital transfer	3 cases
Pediatrics–internal medicine	1 case
Duration of treatment discontinuation (years)	9.5 (4–19)

The data are presented as medians, with minimum and maximum values.

ALB, albumin; ALT, alanine aminotransaminase; AST, alanine aminotransferase; D‐BIL, direct bilirubin; GGT, γ‐glutamyl transferase; K–F ring, Kayser–Fleischer ring; LT, liver transplantation; MELD, model of end‐stage liver disease; NH3, ammonia; PLT, platelet; T‐BIL, total bilirubin; U‐copper, urinary copper; WBC, white blood cell.

**Table 2:** Pathological findings in the liver and outcome

Case	Age (years)	Pathological findings in the liver	Discontinued treatment	Outcome (years)
1	22	Macronodular cirrhosis		Alive (46)
2	63	Portal fibrosis	(+)	Alive (73)
3	27	Micronodular liver cirrhosis		LT (25)/alive (43)
4	24	Steatohepatitis, F0	(+)	Alive (41)
5	28/45	Steatohepatitis F0–1/ F3–4	(+)	Alive (45)
6	31	Liver cirrhosis		Alive (50)
7	42/47	Steatohepatitis F3/ F3		Alive (51)
8		—		LT (21)/died (42)
9	30	Liver cirrhosis	(+)	Alive (32)
10		—		Died (52)
11		—		Alive (32)
12		—		Alive (50)

### Clinical course of WD patients with liver disease based on onset type

**Figure 1:**
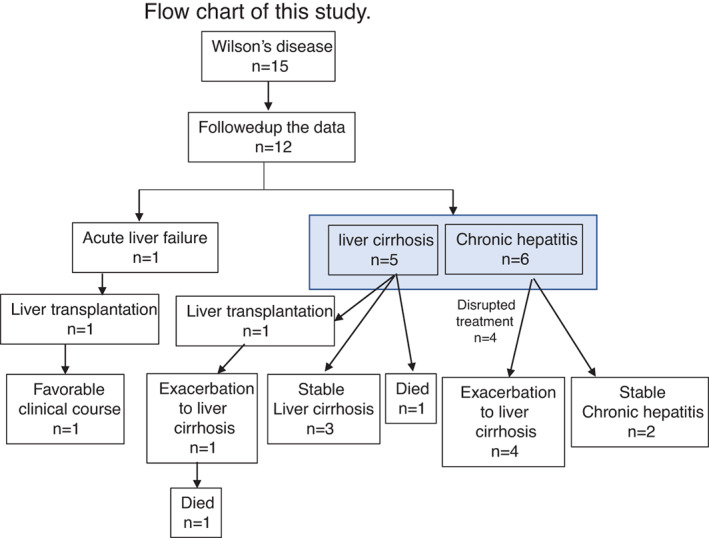
Flow chart of the study patients. The 12 patients (3 male patients and 9 female patients) included in the study had a median age of 28 years (range: 19–57 years) at their first visit to our hospital.

A flow chart describing our patients is shown in Figure 1. The patient with ALF and encephalopathy required LT; her 20‐year postoperative course was good, without the administration of a chelating agent. All 11 patients with CLD began treatment with oral chelating agents, immediately after their diagnosis. Among the six patients with CLD at the time of diagnosis, the cause in four was related to an interruption of treatment, including one patient who was not smoothly transferred from a pediatrics clinic to an internal medicine clinic, and three patients who transferred between internal medicine clinics due to a move. In patient #5, chelation therapy was discontinued for 11 years, which caused liver fibrosis to progress from F0–1 at the age of 28 to F3–4 at the age of 45, as evidenced by a pathological examination (Table 2). The remaining two patients have received continuous treatment and have maintained good health for approximately 30 years.

Liver cirrhosis was present in five patients at the time of diagnosis. One patient underwent LT and received additional treatment with a chelating agent; however, cirrhosis again developed and she died at 21 years after surgery. The 20‐year survival rate after LT was reported to be 66.4% in Japan.^18^ Survival may be lower than in non‐transplant cases. In one patient with liver cirrhosis, progression to liver failure led to a requirement for LT; however, the patient died while waiting for a donor. In this patient, the dose of chelating agent had been reduced due to side effects, such that treatment was suboptimal. The other three patients continue to undergo treatment and their cirrhosis statuses have been unchanged for >10 years. There were no cases of hepatocellular carcinoma (HCC) or hepatic malignancy in any patients.

### Long‐term changes in the levels of noninvasive markers of fibrosis in WD patients with liver disease

**Figure 2:**
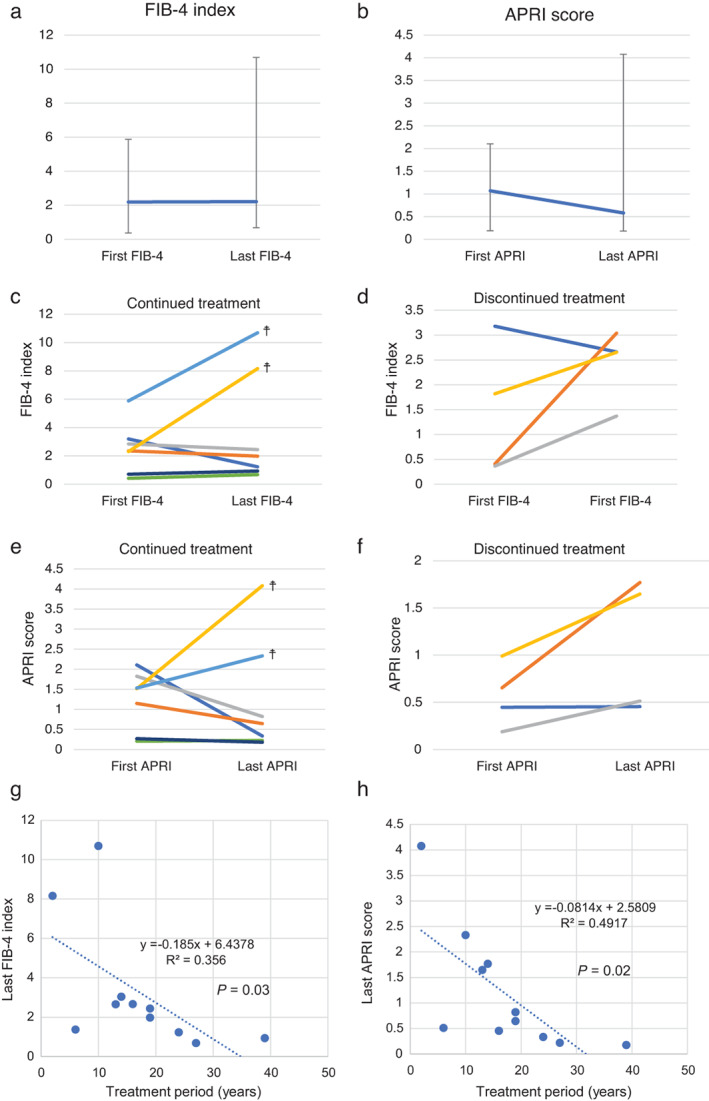
Changes in levels of fibrosis markers in relation to chelation therapy in patients with chronic liver disease (CLD). Changes in the (a) FIB‐4 index and (b) APRI score in WD patients between their first and last visits to our hospital. Changes in the FIB‐4 index (c, d) and APRI score (e, f) of patients with (c, e) continuous and (d, f) interrupted chelating‐agent therapy. Comparison of the duration of chelating‐agent therapy with respect to the (g) FIB‐4 index and (h) APRI score. The fibrosis statuses of the patients were assessed based on the noninvasive determination of fibrosis markers in patients with CLD. At the first visit, the FIB‐4 index and APRI score were 2.20 (0.37–5.89) and 1.07 (0.19–2.11), respectively. The FIB‐4 index (a) increased to 2.21 (0.68–10.69) and APRI score (b) decreased to 0.58 (0.18–4.08) during the follow‐up period, although these changes were not statistically significant. In patients with continuous treatment (*n* = 7), the FIB‐4 index decreased or stable in five patients and liver transplantation was not required (c); conversely, in patients with discontinued treatment (*n* = 4, (d), the FIB‐4 index increased. Similar results were obtained regarding the APRI score and may have contributed to end‐stage liver failure (e, f). The duration of chelation therapy was significantly negatively correlated with the FIB‐4 index (g) and APRI score (h) at the last visit, as shown in the scatter plot (*R* ^2^ = 0.356, *P* = 0.03, and *R* ^2^ = 0.4917, *P* = 0.02, respectively). APRI, aspartate aminotransferase to platelet ratio; FIB‐4, fibrosis‐4, WD, Wilson's disease. †, death or liver transplantation.

All patients underwent monitoring for their fibrosis status based on noninvasive markers of fibrosis in CLD. The FIB‐4 index and APRI score were evaluated at the first and last visits to our hospital. At the first visit, the median FIB‐4 index was 2.20 (0.37–5.89) and the median APRI score was 1.07 (0.19–2.11). The FIB‐4 index (Fig. 2a) increased to 2.21 (0.68–10.69) and APRI score (Fig. 2b) decreased to 0.58 (0.18–4.08) during the follow‐up period, but the changes were not statistically significant. In the seven continuously treated patients, the FIB‐4 index decreased or stable in five patients and LT was not necessary (Fig. 2c); conversely, the FIB‐4 index increased in the four patients with discontinued treatment (Fig. 2d). Similar results were obtained regarding the APRI score, with a decrease in continuously treated patients (Fig. 2e) and an increase in patients with discontinued treatment or end‐stage liver failure (Fig. 2e,f). The duration of chelating‐agent therapy and the FIB‐4 index (Fig. 2g) or APRI score (Fig. 2h) at the last visit were significantly negatively correlated, as indicated in the scatter plot (*R* ^2^ = 0.356, *P* = 0.03, and *R* ^2^ = 0.4917, *P* = 0.02, respectively).

### Prognosis of WD patients with liver disease

**Figure 3:**
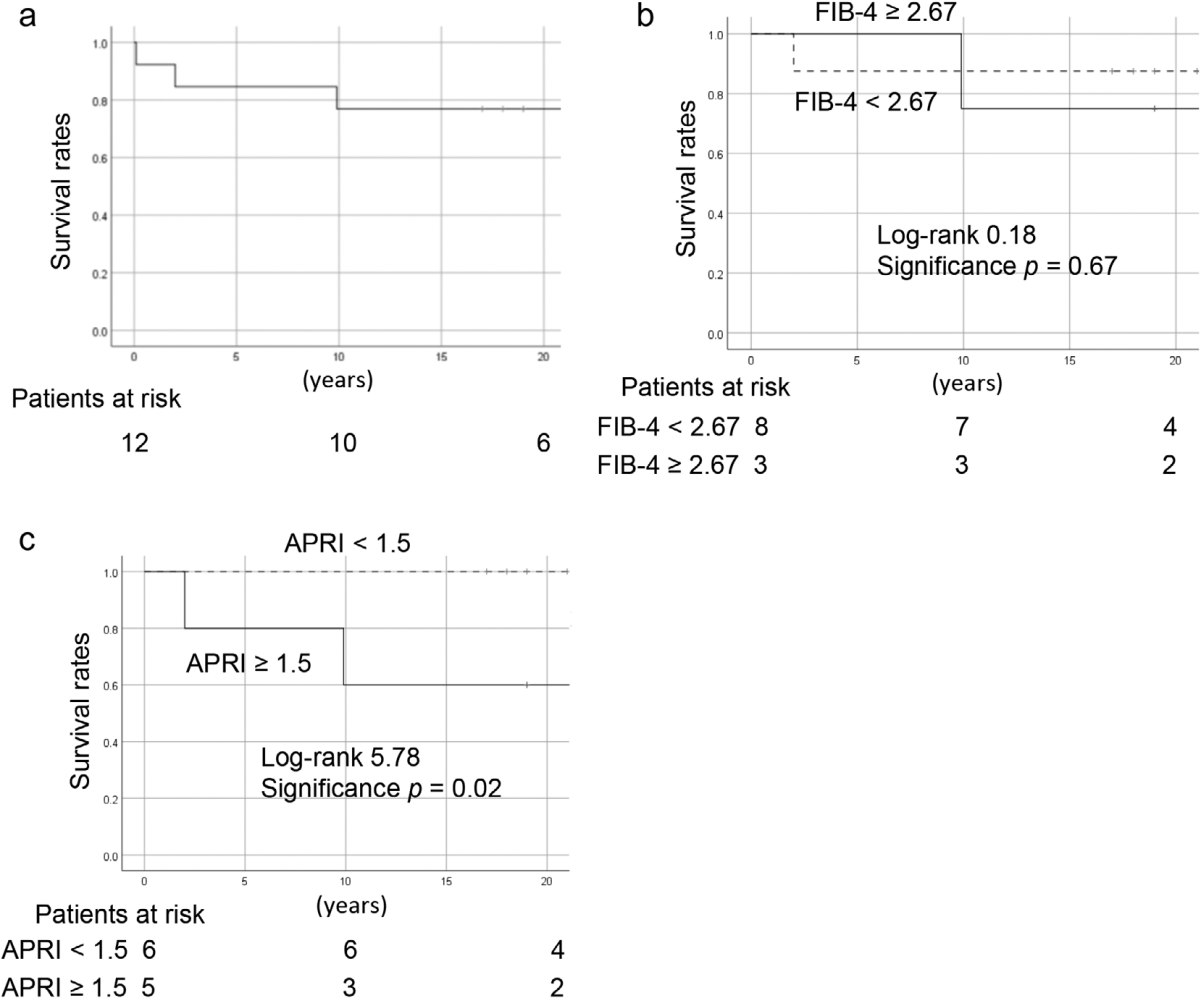
Survival rates of patients with WD. Overall survival as determined from Kaplan–Meier curves (a) and subgroup analyses based on the (b) FIB‐4 index (<2.67 and ≥2.67) and c) APRI score (< 1.5 and ≥1.5). There was no significant difference in patient outcome based on the FIB‐4 index, whereas patients with an APRI score ≥1.5 had a worse outcome than did patients with an APRI score <1.5. APRI, aspartate aminotransferase to platelet ratio; FIB‐4, fibrosis‐4; WD, Wilson's disease.

The overall survival rate of our patients was assessed using Kaplan–Meier curves (Fig. 3a). In the subgroup analysis of survival rates, the difference between patients with an FIB‐4 index <2.67 and patients with an FIB‐4 index ≥2.67 at the first visit to our hospital was not statistically significant (Fig. 3b). However, the survival of patients with an APRI score ≥1.5 was worse than the survival of patients with an APRI score <1.5 (Fig. 3c). Patients with CLD continuously treated with chelating agents had a good clinical course for >10 years, without worsening of liver function or development of a related malignancy. However, in all four patients with disrupted treatment (Table 2), progressive liver worsening was observed, especially in patients #2 and #5, both of whom had discontinued treatment for >10 years. In patient #2, the FIB‐4 index decreased while the APRI score remained stable; in patient #5, both the FIB‐4 index and APRI score were elevated.

## Discussion

The 12 patients (3 male patients and 9 female patients) included in our study were followed‐up for >20 years after their diagnosis of WD. Favorable outcomes were observed in the patient with ALF who underwent LT and the patients with CLD (including patients with cirrhosis) who received chelating therapy. Patients with an APRI score ≥1.5 had a worse prognosis, but this value decreased in response to chelating‐agent therapy. Patients with interrupted treatment developed liver disease. These results demonstrate that the long‐term administration of an oral chelating agent and appropriate management are important for improving the long‐term prognosis of patients with WD and liver disease.

Hepatic involvement in WD tends to begin at a younger age, compared with neuropsychiatric involvement.^19^, ^20^ The majority of the clinical symptoms of WD become apparent in patients who are 5–35 years of age.^1^ In a group of 1223 patients, WD manifested after 40 years of age in only 3.8%, and two‐thirds of these older patients had neurological symptoms.^21^ All of our patients were diagnosed at <40 years of age. In their cohort of 163 WD patients who underwent long‐term follow‐up, Merle et al.^22^ found that patients with neurological symptoms were significantly older at symptom onset than were patients with hepatic symptoms (20.2 *vs* 15.5 years of age, *P* < 0.05); moreover, outcomes were worse among patients with neurological symptoms. Three of our patients, including the patient who died of liver failure (patient #10), had neurological symptoms or disturbed mental functioning.

The majority of our patients (9 of 12; 75%) were girls or women. Ferenci et al.^21^ reported that WD more commonly presented with neurological symptoms in older men, whereas a hepatic presentation was more frequent in women. ALF is also more often observed in girls or women.^23^ The pattern of onset and type of ALF may be associated with survival. Ferenci et al.^24^ examined the age of WD onset in 1357 patients (702 children and 655 adults) and found that 39.5% of children/adolescents (age ≤18 years) and 58% of adults already had cirrhosis at the time of diagnosis. In our patients, five (41.7%) had cirrhosis at diagnosis. Ferenci et al.^24^ also reported that neither laboratory values nor the ATP7B genotype were correlated with cirrhosis. Long‐term follow‐up of our patients showed nonsignificant increases in the FIB‐4 index and APRI score after the first visit to our hospital; however, both values decreased in response to chelating therapy. Therefore, the adequate long‐term treatment of WD may lead to improvements in liver function and fibrosis. We also found that patients with an APRI ≥1.5 had a poor outcome; significant differences were observed with respect to an APRI ≥1.0. Thus, the APRI score may serve as a prognostic indicator in patients with WD.

Treatment with d‐penicillamine improved most of the hematologic and neurologic abnormalities,^7^, ^25^ but it had minimal effects on hepatomegaly and splenomegaly; it did not reverse cirrhosis.^26^ The long‐term survival of WD patients treated with d‐penicillamine was similar to the long‐term survival of age‐ and sex‐matched controls. Penicillamine and zinc are effective in ameliorating the hepatic symptoms in WD, although penicillamine has severe side effects.^27^ A study from Germany demonstrated that 76.1% of WD patients had a stable or improved disease course after treatment initiation.^22^ Other studies have shown that treatment withdrawal is associated with a poor outcome,^28^, ^29^ and some authors have suggested that patients should continue treatment during pregnancy.^30^ Among our patients, four were treated with zinc acetate dihydrate, three were treated with d‐penicillamine, nine were treated with trientine hydrochloride, and four were treated with pyridoxal phosphate hydrate. Six patients had a favorable outcome in response to treatment, whereas progression to liver cirrhosis occurred in the four patients with treatment discontinuation and in the patient requiring LT. Urgent LT has good outcomes for late‐onset presentations and recommend that urgent transplantation should always be considered in WD presenting as ALF.^31^ These results suggest that the APRI score is an indicator of fibrosis in WD, because patients with a longer duration of therapy showed a reduction in their APRI score. Liver function was preserved in our continuously treated patients, as determined over a maximum follow‐up of 39 years (median: 20 years), which suggests that chelating therapy reduces fibrosis progression in patients with WD complicated by liver disease, including patients with cirrhosis. None of our patients developed HCC. The prevalence of hepatobiliary malignancies was reported in a cohort of 1186 patients^32^; only 1.2% developed a hepatobiliary malignancy (eight patients with HCC and six patients with intrahepatic cholangiocellular carcinoma), corresponding to an incidence of 0.28 per 1000 person‐years.

Patients with WD and end‐stage liver failure are candidates for LT. A prognostic index for WD was modified by Dhawan et al.^15^ to allow determination of the optimal timing of LT. In that study, all WD patients with a prognostic index score >11 died without LT.^15^ In our patients with ALF and end‐stage liver failure, the prognostic index scores were high. The ALF patient underwent LT and has had a good 20‐year postoperative course without the administration of a chelating agent.

We reported the survival rates of NAFLD patients previously; 44 patients (12.1%) died during a median of 7.1‐year follow‐up and the overall 10‐year survival rate was 86.2%.^33^ In age‐ and sex‐matched cohorts, the outcome of NASH patients was better than that of hepatitis C virus (HCV)‐related cirrhosis patients.^34^, ^35^ The 10‐year complication‐free survival rate was 48% in NASH patients and 34% in untreated HCV patients.^34^ Although patients with WD had steatohepatitis, their survival rate was better than that of NAFLD patients. The likelihood of HCC was higher in NASH and HCV patients than WD patients.

The limitations of our study included its small sample size and the lack of a pathological examination for some of the patients. Nonetheless, this study of patients with WD showed that interrupted treatment (4 patients, 33.3%) or an insufficient dose of chelating agent in CLD (one patient, 8%) resulted in worsened liver disease. In contrast, patients with CLD who received chelation therapy exhibited good long‐term survival, without a decline in liver function or the development of a malignancy. LT was successful in patients with ALF and end‐stage liver failure. These results demonstrate the importance of continued treatment and indicate that treatment disruption for any reason should be avoided.

### Human and animal rights

All procedures were performed in accordance with the ethical standards established in the 1964 Declaration of Helsinki and its later amendments.

### Informed consent

Informed consent was obtained from all included patients for the publication of this case study.

## Supporting information


**Table S1.** Baseline characteristics and biochemical data of the 12 study patients with Wilson's disease at first visit or diagnosis at our hospital.
