# Supplementary material for: Long‐term outcome of Wilson's disease complicated by liver disease
Source: JGH Open. 2021 Jun 5;5(7):793–800. doi: 10.1002/jgh3.12589 (PMC8264237; doi:10.1002/jgh3.12589)
Supplement: Supplementary file 1 — Table S1. Baseline characteristics and biochemical data of the 12 study patients with Wilson's disease at first visit or diagnosis at our hospital. [file JGH3-5-793-s001.docx]

Supplemental Table 1 Baseline characteristics and biochemical data of the 12 study patients with Wilson’s disease at first visit or diagnosis at our hospital.

| Case | 1 | 2 | 3 | 4 | 5 | 6 | 7 | 8 | 9 | 10 | 11 | 12 |
| --- | --- | --- | --- | --- | --- | --- | --- | --- | --- | --- | --- | --- |
| Age at diagnosis (years) | 22 | 38 | 25 | 23 | 28 | 31 | 32 | 18 | 11 | 42 | 5 | 11 |
| Age at referral to our hospital (years)* | 22 | 57 | 25 | 23 | 23 | 31 | 32 | 19 | 30 | 52 | 24 | 50 |
| Duration from diagnosis to last observation (years) | 24 | 35 | 18 | 18 | 17 | 19 | 19 | 24 | 21 | 10 | 27 | 39 |
| Sex (M, male; F, female) | F | M | F | M | F | F | F | F | F | F | F | M |
| Acute (A)/chronic (C) | C | C | A | C | C | C | C | C | C | C | C | C |
| K–F ring |  | (+) | (+) |  | (+) | (+) |  | (+) | (+)** | (+) |  |  |
| Neuronal symptoms | (+) | (+) |  |  |  |  |  |  |  |  |  |  |
| Psychiatric symptoms | (+) |  |  |  |  |  |  |  |  | (+) |  |  |
| Esophageal varices |  | (+)** |  |  |  |  |  | (+)** | (+)** | (+) |  |  |
| Familial onset |  | Brother | Sister | Brother | Sister | Sister | Sister | Sister, Brother |  |  | Sister | Sister |
| Initial diagnosis of liver cirrhosis | (+) |  |  |  |  | (+) | (+) | (+) |  | (+) |  |  |
| **Biochemical analysis at *** |  |  |  |  |  |  |  |  |  |  |  |  |
| ALB (g/dL) | 2.8 | 4 | 3.2 | 4.3 | 3.9 | 3.5 | 2.8 | 3.4 | 3.1 | 1.9 | 4.4 | 4.8 |
| T-/D-BIL (mg/dL) | 1.6 | 0.6 | 40.8/20.3 | 0.6/0.3 | 0.2 | 1.3/0.7 | 1.2/0.7 | 1.6 | 0.6 | 7.9/3.0 | 0.5 | 0.6 |
| AST (U/L) | 89 | 18 | 107 | 49 | 15 | 33 | 65 | 19 | 34 | 44 | 15 | 20 |
| ALT (U/L) | 23 | 7 | 36 | 135 | 15 | 25 | 46 | 17 | 29 | 20 | 16 | 39 |
| GGT (U/L) | 188 | 17 | 63 | 58 | 55 | 66 | 124 | 19 | 116 | 91 | 15 | 43 |
| Copper (μg/dL) |  |  | 214 | 23 | 20 | 31 | 34 | 5 | 27 | 30 |  | 23 |
| Ceruloplasmin (mg/dL) |  |  | 14 | 2 | 5 | 3 | 3 | < 2.0 | 11 | 6 | ≤ 2 | 6 |
| U-copper (μg/day) |  |  | 14,965 | 113.5 |  | 102 | 606.9 | 131.8 | 102 | 573.8 | 2 | 880 |
| NH3 (μg/dL) |  |  | 45 |  |  | 10 |  | 109 |  | 79 |  | 22 |
| PT (%)/INR | 47.8/1.58 |  | 45.6 | 99.6/1.00 |  |  | 54.4/1.41 |  | 57.7/1.29 | 28.9/2.01 | 88.6/0.99 | 100/0.90 |
| WBC (/μL) | 3,690 | 3,020 | 14,900 | 4,840 | 5,000 | 3,850 | 6,780 |  | 3,830 | 9,030 | 4,630 | 4,860 |
| PLT (×10^4^/μL) | 19.9 | 15.3 | 25.7 | 11.3 | 17.1 | 15 | 15.5 | 5.2 | 6.8 | 2.6 | 17.6 | 22.0 |
| MELD score | 10 | 3 | 28 | 2 | 6 | 5 | 10 | 5 | 1 | 22 | 13 | 3 |
| Prognostic index | 3 | 1 | 11 | 1 | 1 | 1 | 3 | 1 | 2 | 10 | 1 | 0 |
| **Treatment** |  |  |  |  |  |  |  |  |  |  |  |  |
| Zinc acetate dihydrate |  |  |  | (+) | (+) |  |  | (+) | (+) |  |  |  |
| Trientine dihydrochloride | (+) | (+) |  | (+) | (+) | (+) | (+) | (+) | (+) | (+) |  |  |
| d-penicillamine |  |  |  |  |  |  |  |  |  | (+) | (+) | (+) |
| Pyridoxal phosphate hydrate |  |  | (+) |  | (+) | (+) |  |  |  | (+) |  |  |
| Discontinued treatment |  | (+) |  | (+) | (+) |  |  |  | (+) |  |  |  |
| Self-interruption of treatment |  | (+) |  | (+) | (+) |  |  |  | (+) |  |  |  |
| Length of discontinuation  Hospital transfer |  | (+) |  | (+) | (+) |  |  |  |  |  |  |  |
| Pediatrics–internal medicine |  |  |  |  |  |  |  |  | (+) |  |  |  |
| Duration of treatment discontinuation (years) |  | 19 |  | 4 | 11 |  |  |  | 8 |  |  |  |

** observed during follow-up. ALB, albumin; ALT, alanine aminotransaminase; AST, alanine aminotransferase; D-BIL, direct bilirubin; GGT, γ-glutamyl transferase; K–F ring, Kayser–Fleischer ring; LT, liver transplantation; MELD, Model of End-Stage Liver Disease. NH3, ammonia; PLT: platelet; T-BIL, total bilirubin, U-copper, urinary copper; WBC: white blood cell.
